# Challenges in delivering nutrition care perceived by hospital dietitians in the Czech Republic: a qualitative study

**DOI:** 10.1136/bmjopen-2025-101787

**Published:** 2025-11-04

**Authors:** Martin Krobot, Kamila Jančeková, Victoria H Hawk, Veronika Zelenková, Zlata Kapounová

**Affiliations:** 1 Department of Public Health, Faculty of Medicine, Masaryk University, Brno, Czech Republic; 2 Department of Nutrition, The University of North Carolina, Greensboro, North Carolina, USA

**Keywords:** QUALITATIVE RESEARCH, Interprofessional Relations, Delivery of Health Care, Integrated, Hospitals

## Abstract

**Abstract:**

**Objectives:**

Nutrition care is a crucial component of healthcare, with dietitians playing a key role in patient outcomes. However, their integration into interdisciplinary teams remains limited. While there are countries where dietitians are well situated, in the Czech Republic, the profession of a dietitian was established in 2004 and is still undergoing a transformation. Hence, this study aims to explore the experiences and challenges in delivering nutrition care that Czech dietitians encounter while working in a hospital setting.

**Design:**

Data were collected through semistructured interviews with hospital dietitians. Participants were recruited purposefully via professional associations, social media and chain referrals. Interviews were transcribed and analysed using applied thematic analysis with an inductive coding approach.

**Setting:**

Interviews were held with hospital dietitians from across the Czech Republic. All interviews were conducted online via ZOOM.

**Participants:**

A total of 15 hospital dietitians took part in the study, predominantly female, covering a wide range of formal level of dietetics education, years of practice and professional roles.

**Results:**

Three main themes were identified: (1) unclear professional role and identity, (2) marginalisation of nutrition care and (3) tension between professional aspirations and legal boundaries. The professional identity of dietitians remains fragmented, especially in the matter of what constitutes the role of a dietitian. Participants reported limited recognition by other healthcare professionals and insufficient organisational support. Barriers such as inadequate staffing, lack of autonomy and absence of strategic prioritisation of nutrition care were perceived as key obstacles to effective interprofessional collaboration and delivery of nutrition care.

**Conclusions:**

Hospital dietitians in the Czech Republic face systemic, organisational and team-based challenges that may limit their ability to deliver effective nutrition care. The findings suggest that clarifying professional roles, improving awareness among healthcare staff and exploring supportive institutional practices could help strengthen their integration into interprofessional teams. Further research is needed to examine how changes in educational structures, staffing policies and management approaches would influence the delivery of nutrition care and the evolving role and professional identity of dietitians.

**STRENGTHS AND LIMITATIONS OF THIS STUDY**

- This study employed applied thematic analysis using semistructured interviews, allowing for in-depth exploration of hospital dietitians’ experiences and perceptions across various healthcare settings in the Czech Republic.
- Purposive sampling with maximum variation ensured inclusion of dietitians with diverse educational backgrounds and types of hospitals, enhancing the transferability of findings.
- Data collection and analysis were conducted by a team including both experienced researchers and practising dietitians, increasing the credibility and contextual understanding of the results.
- As the research focused solely on dietitians’ perspectives, it does not capture the views of other healthcare professionals, which could provide a more comprehensive understanding of interdisciplinary collaboration in nutrition care.

## Introduction

 Hospital malnutrition is one of the prominent factors affecting the prognosis of a patient, impacting on up to 65% of patients.^1^ If left untreated, patients can suffer from impaired wound healing, increased risk of infections or complications, and overall increased morbidity and mortality.^2^ In 2023, the European Society for Clinical Nutrition and Metabolism (ESPEN) issued the Vienna declaration, stating that nutrition care is a human right and an integral component of healthcare.^3^ Subsequently, the European Federation of the Associations of Dietitians (EFAD) issued the Budapest resolution, stating that dietitians play a pivotal role in delivering nutrition care and improving patient outcomes as healthcare professionals (HCPs) educated in food and nutrition and members of interprofessional teams.^4^

EFAD defines dietitians as ‘recognised healthcare professionals, educated to at least bachelor level’, who ‘assess specific nutritional requirements throughout the life span and translate this into advice and/or treatment’ in clinical, food service or community area.^5^ However, although interprofessional collaboration and education are supported worldwide with WHO issuing a framework on these already in 2010, the role and competencies of dietitians often remain underappreciated among other HCPs, limiting the integration of dietitians into interprofessional teams,^6^ and nutrition remains underrepresented in medical curricula. Current studies show that medical students often feel unprepared to provide nutrition care and lack awareness of the role of dietitians in clinical practice.^7 8^

The position of dietitians also differs worldwide. In countries like the UK, allied health professionals (AHPs) have long been integrated into clinical care pathways. They benefit from a clear scope of practice defined by the Health and Care Professions Council, and they are increasingly recognised as autonomous professionals contributing to multidisciplinary decision-making and service development.^9^ However, in the Czech Republic, dietetics does not have such a strong position or history.^10^

Czech dietitians were officially recognised in 1951 as dietary nurses, with a job description comprising only food service roles under medical supervision. A major turning point came no earlier than 2004, when new legislation introduced dietitians as distinct AHPs with university-level training pathways. However, despite this formal recognition, the scope of practice defined by the law has remained limited with no decision-making authority or oral nutrition supplements (ONS) prescription rights, and the current workforce includes practitioners with vastly different educational backgrounds in dietetics (high school or college before 2004; college, BSc or MSc after 2004), sharing the same professional title and responsibilities.^10^ Furthermore, there is no minimal standard for staffing, resulting in 8.05 dietitians employed in healthcare facilities per 100 000 population, which is significantly lower compared with the USA or UK.^11^ Dietitians are also represented by two separate professional bodies established in 1992 and 2018, lacking one strong supportive voice.^10^

Although factors influencing interprofessional collaboration in healthcare are well described,^12^ little is known about how this applies especially to dietitians in countries with a relatively recent development of AHPs such as the Czech Republic. Existing studies are mostly focused on countries where dietetics have a rich history, such as Canada^13^ or the USA.^14^ This study, therefore, aims to explore the challenges in delivering nutrition care faced by Czech dietitians working in a hospital setting. By using applied thematic analysis (ATA), we seek to better understand the interplay between professional identity, systemic constraints and interprofessional collaboration barriers, aiming to inform future research and implementation efforts aimed at strengthening the role of dietitians within the healthcare system.

## Methods

### Study design

This qualitative cross-sectional study followed the ATA methods outlined by Guest *et al*^15^ and adheres to the Consolidated Criteria for Reporting Qualitative Research.^16^ The methodological framework of ATA combines components of grounded theory and phenomenology in a positivist and interpretive manner. ATA is focused on a transparent and credible process; hence the positivist epistemological approach requiring all assertions to be based on evidence. Interpretive approaches are also available to analyse the meanings, understanding and behaviour of participants.^15^

### Recruitment

We used maximum variation purposive sampling^17^ to recruit hospital dietitians from the Czech Republic based on predefined criteria regarding their level of education in dietetics (high school, college, BSc, MSc) and the type of hospital they worked in (regional, university, specialised). Participants for piloting the data collection were identified through professional contacts and invited for pilot interviews. Subsequently, the study was advertised via professional associations and social media. At the end of each interview, participants were asked whether they knew of other dietitians who might meet the predefined criteria, allowing for additional recruitment via chain referral.^17^ No incentives were provided to motivate participation. While some participants were known to the interviewer, they responded to the advertisement of their own will and were not invited based on their relation to the interviewer except for the two initial pilot participants. Since the study aim was stated clearly in the advertisement, it is possible that the sample was skewed towards dietitians who were more dissatisfied or had experienced more challenges in their workplace. This potential self-selection bias was considered in the analysis and results interpretation.

Informed consent was obtained from all participants before scheduling the interview. The entire research team signed confidentiality agreements to ensure data protection and prevent potential data breaches, including the assistants hired for interview transcription.

### Data collection

We conducted online, in-depth, semistructured interviews using conversational interviewing via the ZOOM platform to engage dietitians from across the country. The interview guide (table 1; full version in online supplemental file 1) consisting of open-ended questions was developed in consultation with a senior researcher and a nutrition practitioner expert and then face-validated with three dietitians not involved in the study. The first two interviews were used to pilot the data collection process. Since there was no need for a change in the process, these interviews were included in the dataset.

**Table 1:** Domains of the interview guide and example questions

Domain	Example question
Job satisfaction	What do you enjoy, find fulfilling about your work?
Perception by other health professionals	How do you feel involved in the interdisciplinary team at your workplace and what is your role in it?
Perception of job description	To what extent does your job description match your expectations?
Perception of nutrition care	What are the barriers in your workplace that prevent you from providing care as you would like to?

All interviews were conducted between May 2021 and February 2022 in Czech by MK to ensure consistency. MK is a registered dietitian and an assistant professor at a university dietetics programme. At the time of the study, he was also serving as the chair of the Czech Association of Dietitians. While this background provided valuable contextual understanding and rapport with participants, it may have also influenced the responses due to perceived professional authority or social desirability, which was reflected during the interviews and the subsequent analysis. Participants were informed about the researcher’s role before the interview, and efforts were made to create an open and non-judgemental atmosphere during the interviews to minimise the effect on participants’ responses.

The interviews lasted between 21 and 71 min (median length 36 min). A notetaker (ZK or VZ) was present in each recorded part of the interview to record field notes in order for the interviewer to focus fully on the interview^18^ and also as a method of triangulation.^19^ We acknowledge that even an unseen observer may influence participants’ comfort and willingness to share sensitive information. To minimise this potential impact, notetakers were introduced under pseudonyms, did not interact with participants and remained off-camera throughout the interview. None of the participants expressed concerns about the presence of a second researcher before, during or after the interview.

To protect the participants’ data, only the principal investigator knew their identity and characteristics. Each participant was assigned a code, which was used to pseudonymise all the materials related to that participant and under which they were joining the ZOOM call with their camera turned off, and the participants were also advised to avoid disclosing names of people or places during the interview. Demographic data had been acquired before the recorded part of the interview and before the notetaker joined the call. No repeat interviews were carried out. The audio of each interview was recorded and transcribed by research assistants using oTranscribe. All transcriptions were compared with the recordings for accuracy and readability and de-identified by MK before analysis.

### Data analysis

Thematic analysis followed an inductive approach, grounded in the principles of ATA described by Guest *et al*.^15^ All interview transcripts were analysed alongside field notes. Two researchers (MK and KJ) independently conducted open coding of the transcripts to capture recurring patterns and concepts. An initial codebook was created and iteratively refined through team discussions, ensuring both consistency and flexibility during the coding process. Final coding was performed using Atlas.ti V.23 (ATLAS.ti Scientific Software Development, Berlin, Germany).

Once coding was completed, codes were reviewed for conceptual similarities and grouped into preliminary categories. Through a series of collaborative meetings involving both academic researchers and practising dietitians (MK, KJ, VZ, ZK and VHH), these categories were further organised into candidate themes and subthemes. The process included constant comparison of coded segments across transcripts, identification of patterns and discussion of potential overlaps. Special attention was paid to maintaining internal coherence within each theme and ensuring distinct boundaries between themes.

The final thematic structure was agreed on through consensus among all members of the research team. During the peer-review process, the structure was revisited in response to reviewer feedback, prompting regrouping and renaming of some themes to better reflect the research aim and reflect the introduction of theoretical frameworks for interpretation. Throughout the analysis, we aimed to preserve participants’ voices while situating their perspectives within a broader institutional and professional context.

Although the thematic analysis was conducted inductively, the interpretation of the findings was subsequently informed by two theoretical frameworks. On the level of the profession, the concept of professionalism by Evetts^20^ distinguishes between occupational and organisational professionalism, with the first one coming from within the community (eg, trust, ethics, professional associations) and the latter from the outside (eg, guidelines, regulation, managerialism). Furthermore, since dietitians as AHPs should be integrated into interprofessional teams, the systematic meta-review of Wei *et al*^12^ proposes a three-level framework for the classification of barriers to interprofessional collaboration into individual, team and organisational. These frameworks were used to interpret the themes and discuss their broader implications for the professionalisation of dietitians in the Czech Republic and the delivery of nutrition care within hospitals.

### Patient and public involvement

Only dietetic professionals were included in the study; therefore, the study did not involve any patients or the public.

## Results

Several hundred dietitians were approached online, and 11 dietitians were approached via chain referrals from previous participants. Out of these, 26 expressed an interest to participate, of which 15 completed informed consent and participated in the research. Overall, the participants represented dietitians aged 24–63, mainly female, and working in a variety of hospital settings, including large and small hospitals at clinical or food service positions and outpatient clinics. Participants comprised dietitians with different levels of education and varied amounts of professional experience in several types of hospitals, thus offering multiple perspectives and expertise. Their characteristics are summarised in table 2.

**Table 2:** Participant characteristics

		Number	%
Sex*	Male	2	13
	Female	13	87
Age	24–30	6	40
	31–50	7	47
	51–63	2	13
Years of practice	0.25–10	8	53
	11–20	3	20
	21–44	4	27
Highest level of formal dietetics education	High school	4	27
	College	3	20
	University (BSc)	4	27
	University (MSc)	4	27
Type of hospital	Specialised hospital	2	13
	University hospital	6	40
	Regional or city hospital	7	47
Professional role†	Inpatient care	13	87
	Outpatient care	10	67
	Food service	8	53
	Chief dietitian	2	13

* The term ‘sex’ refers to biological sex as reported by participants during demographic data collection. In the original Czech version of the interview guide, the term ‘pohlaví’ was used, which corresponds to sex assigned at birth. No data on gender identity were collected.

† Participants could report more than one professional role. Therefore, the percentages account for the share of all the participants claiming that role and do not sum to 100%.

Through thematic analysis, we identified three themes and four subthemes, which are further described below (see table 3). Although the interviews and analysis were held in Czech, we translated the quotes for the purpose of reporting the research.

**Table 3:** Themes and subthemes

Theme	Subtheme
Unclear professional role and identity	Insufficient recognition of the dietitian’s role
	Internal fragmentation of professional identity
Marginalisation of nutrition care	Lack of strategic support for nutrition care
	Disregard of nutrition care by other healthcare professionals
Tension between professional aspirations and legal boundaries	

### Theme 1: unclear professional role and identity

#### Subtheme 1.1: insufficient recognition of the dietitian’s role

Dietitians frequently described misalignment between how they saw their role in healthcare and how it was perceived by other HCPs. While some participants identified instances of respectful collaboration, most of them reported being associated only with food service. This resulted in inferior treatment compared with other clinical HCPs, including a lack of respect and exclusion from interprofessional collaboration or the assignment of irrelevant tasks. Attempts to justify their position and emphasise the importance of nutrition care were often perceived as futile. Dietitians attributed this phenomenon especially to the lack of awareness about dietitians’ education and scope of practice among other HCPs.

My colleague’s ward nurse actually claimed that she’s not even a healthcare professional. So yeah… the awareness is really, really poor. (…) Before, there were just the ‘dietaries’ in the kitchen, so now they see me as just another ‘dietary’ who suddenly showed up on the ward. But no one really knows what I’m supposed to be doing or how it’s supposed to work. D13, MSc, regional hospital

This issue was individualised with dietitians sharing instances of both refused and embraced collaboration, even at the same hospital, and the influence of the opinion of a respected figure leading the team (eg, head physician, charge nurse) on the behaviour of the whole department in a positive or negative way. They also pointed out that generational factors seemed to play a role, describing younger physicians as more open to collaboration, and large hospitals as more likely to foster professional recognition compared with smaller regional hospitals.

We had this hugely respected chief surgeon who openly claimed that dietitians had no place in a hospital, that they were completely useless. And over the course of thirty years, he basically manipulated the whole hospital into believing that. D09, BSc, regional hospital

Although participants described situations where collaboration was facilitated by other professionals’ positive experiences with dietitians—particularly their expertise, clinical reasoning and interpersonal skills—these strategies were not always effective. Many expressed frustration that in some settings, professional recognition seemed impossible to reach regardless of effort, which could indicate the presence of other barriers to integration. Interestingly, some participants named physiotherapists to have a more established position, noting that other HCPs understood the physiotherapists' role and contributions to the team and perceived their legal professional status as superior.

I started out on the surgical ward, and I just couldn’t handle it mentally with them. So, I asked to be transferred. I was really interested in it, the patients, the fact that nutrition plays a huge role there, especially with surgical patients and obviously in GI cases, but it just wasn’t doable. The communication there was really bad. D01, MSc, university hospital

#### Subtheme 1.2: internal fragmentation of professional identity

In addition to reporting a lack of awareness among other HCPs, participants expressed diverse views on their own professional role and identity. Their understanding of the nature of the dietitians’ job description was fragmented, and the perception about the necessary level of dietitians’ education differed.

These differences were most evident in how participants were talking about both the clinical and operational dimensions of their work. While some considered involvement in food service management to be integral to their role, highlighting benefits of combining food service and clinical dietetics in one position, others saw it as unrelated or even detrimental to how the profession is perceived by other HCPs. Similarly, perceptions of what constitutes clinical practice varied; for some, clinical dietetics was understood primarily as pragmatic care focused on food-based interventions, while others emphasised the dietitian’s role in ONS prescription and specialised nutrition care. These perspectives not only reflected differences in individual experience, education and training, but also revealed the absence of a shared understanding of the profession’s core focus and role in healthcare.

There’s this typical attitude: ‘I’m not going to work in the kitchen.’ But I’m lucky that in our hospital, the role is combined. D14, college, regional hospital

Changing the bread for the bun, that’s the job of the… dietary nurse? Or they call themselves dietitians… I think there should probably be some kind of distinction. Because when the dietitian from the kitchen picks up the phone, she introduces herself as a dietitian, and I also introduce myself as a dietitian, and then I can imagine that some doctor here really has no idea who’s who. D01, MSc, university hospital

These conflicting viewpoints are not only limited to the simple description of a dietitian’s role but also subvert the unity of the profession. Participants tend to perceive their understanding of the dietitian’s role as the right one and criticise different opinions of others from this perspective. University-educated participants expressed unfairness about sharing the same scope of practice with high school graduates, voicing that their inadequate training for clinical practice might be harming the profession’s reputation among other HCPs. Similarly, dietitians based in food service raised concerns about dietitians or students with university education and training, pointing out their lack of experience and misconceptions about food service.

I think that the university is far more difficult, but then we have it, the scope of practice, the same, or the things we can do let’s say with bachelor’s degree compared to them with their college degree. D04, BSc, regional hospital

And then I see limits in the university education (…) and that’s cooking. To be able to think about the composition of a diet, replacements, creating new recipes, food plan adjustments (…) that’s what I almost can’t find in university educated dietitians, bachelor or master, who didn’t graduate from dietetic high school before. D02, high school, university hospital

Despite being internally divided, participants across the spectrum perceived insufficient support and representation from professional organisations, whose division they understood as a barrier to advancing the profession. This tension between internal divergence and external expectations further highlights the unsettled nature of dietitians’ professional identity.

I think that the projects are often similar in both organizations, and that sometimes we’re just unnecessarily dividing our efforts. D05, college, regional hospital

### Theme 2: marginalisation of nutrition care

#### Subtheme 2.1: lack of strategic support for nutrition care

Many participants addressed the persistent marginalisation of nutrition care by the hospital management. Proposals regarding allocating resources, new equipment, systemic changes or opening new positions for dietitians were usually dismissed or deprioritised. Although economic argumentation was used frequently by the management, nutrition care was rarely considered a strategic priority, being perceived as peripheral and unprofitable. Dietitians viewed this as a fundamental contradiction, noting that effective nutrition interventions could improve patient outcomes while reducing hospital costs. In their view, the persistent deprioritisation reflected a lack of understanding of both the clinical and economic value of nutrition interventions.

[The chief dietitian] is desperate because of the 55 CZK [for food per day; cca 2,20 EUR]. He applied for an increase in the food budget, since apparently it hasn’t been updated since 1992. And he was told that it definitely would not be increased for at least another three years, because they have other priorities. D14, college, regional hospital

This misunderstanding had a significant impact on the daily work of dietitians and their ability to deliver evidence-based nutrition care. A majority of participants reported being understaffed, leading to cutting the time spent with the patients or prioritising only the most critical nutrition concerns. Many of these participants spoke about working overtime or after hours at home, and investing their free time into professional development, voicing their concerns about professional burnout. Technological infrastructure was also described as inadequate and unlikely to change, with reliance on paper documentation, lack of access to patient records and minimal integration of nutrition care into electronic health systems. However, advocating for even minor improvements in the system was often futile. Participants described all these working conditions as demotivating and disempowering, expressing a sense of helplessness.

Then you can’t even afford to go see a doctor if you have a toothache, because you know that the moment you’re gone, the other [dietitian] will have to take the hit [take your workload]. D11, high school, regional hospital

We don’t have any patient records, no files for the patients who go through our outpatient clinic, it’s basically just on trust. We don’t even have the option to create their folders on the computer, nothing. D14, college, regional hospital

In addition, dietitians mentioned that food service was often neglected, not being considered a part of essential healthcare or having any impact on the patient’s status. As a result, decisions about food service were made without consulting dietitians, further deepening their professional exclusion while also leading to suboptimal food quality.

In the reconstruction [of the food service facilities] somebody plans it from the technical department, and then when you say [what you really need and why], they say, ‘We didn't think of that at all.’ Because they think, ‘We all cook at home, what’s the big deal?’ D05, college, regional hospital

#### Subtheme 2.2: disregard of nutrition care by other HCPs

Another aspect of marginalisation came from the perceived lack of nutrition knowledge among other HCPs. Participants reported this was crucial in shaping the current state of nutrition care and also partly influencing the interprofessional relationships in the team. The insufficient understanding resulted in a lack of interest, often dismissing nutrition as irrelevant or deprioritising it. Consequently, physicians were disregarding the patient’s nutrition status and needs, nurses did not administer sip feeds or perform any nutrition screening, and food service staff did not follow any guidance on food quality or technology, emphasising their cooking experience and cost-saving measures. Participants understood this lack of knowledge as a complex root for most of the issues they encountered in their daily work lives.

…there’s simply no one there, no one with the specialization [in clinical nutrition], or even just with an interest in nutrition. Honestly, sometimes there’s just no one to be found who cares [about nutrition]. D01, MSc, university hospital

In some places, they don’t do any screening at all. They won’t call the dietitian even for a patient who’s thin, has lost weight, isn’t eating, they just don’t call the dietitian, they don’t deal with [nutrition]*.* D08, college, university hospital

### Theme 3: tension between professional aspirations and legal boundaries

Some challenges raised by the participants were not attributable to individual interactions but were more linked to the settings of the system in terms of definition of the profession and related legislative conditions. The current setting appeared not to be matching dietitians’ conceptions of both nutrition care and their corresponding professional role. In this context, dietitians described frustration and concerns about proper delivery of nutrition care.

These systemic barriers manifested in several ways, most notably in compensation, staffing and scope of practice. Participants voiced insufficient salaries in public hospitals, some seeking additional employment or contracts to secure a living. They partly blamed nationally regulated pay grades, but also mentioned management reluctance to raise their salaries, seeing potential link to the absence of reimbursement of dietetic care in hospitalised patients from the insurance companies, making them look unprofitable.

I’m being saved by the student scholarship, plus some grants I’m working on, so overall I’m happy, but if I had just the salary, I don't think [it would be enough]. D12, MSc, university hospital

In terms of scope of practice, participants described frustration from being unable to make even routine decisions independently, such as modifying texture or amount of patient’s food, without physician’s approval. This lack of autonomy led to perceived inferiority, with some participants even considering the constant need for physician’s approval to be one of the causes for misperceptions about dietitians’ competence. Nevertheless, dietitians admitted that a broader scope of practice would require a higher number of dietitians, calling for national guidelines setting minimal staffing limits.

And constantly knocking on [the doctor’s] door to ask if I can get two extra kefirs for some lady from who-knows-where, I honestly feel completely ridiculous. (…) The doctor often doesn’t even know what types of diets exist, and I’m the one who has to beg just to get a kefir prescription. D01, MSc, university hospital

Dietitians frequently called for an expanded scope of practice, advocating for the prescription of ONS or adjusting patients’ diet orders, where they felt better equipped than physicians. Some participants, mostly with master’s degrees, also expressed interest in being authorised to prescribe enteral and/or parenteral nutrition and adjust pharmacotherapy related to nutrition. This vision was not shared by all the participants, with a few expressing a reluctance to take on higher responsibility. However, the contrast of prevalent desire for broader scope of practice and absence of autonomy reflected the broader misalignment between the profession’s aspirations and the structural limitations of the system in which dietitians operate.

Ideally, I’d like to be able to prescribe oral nutritional supplements based on what we consider appropriate. Meaning I’d prescribe the specific products I personally find suitable for that particular patient. D15, BSc, regional hospital

Dietitians may want greater professional authority, but that also means greater responsibility, and I wouldn’t want to be in the doctor’s position regarding responsibility. D02, high school, university hospital

## Discussion

To our knowledge, this is the first qualitative study of Czech dietitians in hospital settings exploring the experiences and challenges in delivering nutrition care they face. The three main themes identified were unclear professional role and identity, marginalisation of nutrition care and tension between professional aspirations and legal boundaries. Overall, the challenges described by participants reflect a complex interplay between underdeveloped organisational professionalism and multilevel barriers to interprofessional collaboration, as conceptualised by Evetts^20^ and Wei *et al*.^12^

### Professional role and identity: who is a dietitian?

The professional identity of Czech dietitians appears to be shaped mainly by occupational professionalism,^20^ including the establishment of university-level education or professional organisations. However, these efforts are undermined by insufficient development of organisational professionalism, including limited autonomy, lack of organisational support or missing national professional guidelines. The existence of multiple professional bodies, divergent educational backgrounds and inconsistent perceptions of the dietitian’s role may then lead to internal fragmentation and hinder the development of a unified professional identity needed to foster the development of the profession, similar to conclusions of Feerick’s study of paramedics.^21^

Clarity of the roles of all the team members is one of the crucial factors of interprofessional collaboration on both the individual and team level.^12^ However, there is still a lack of understanding of the dietitians’ role among HCPs. While some participants acknowledged positive attitudes towards their presence in interprofessional care teams, many reported being disrespected, underutilised and viewed as food service workers. In a Canadian research study, dietitians claimed that the professional acknowledgement from other HCPs is critical for effective nutrition care.^13^ The lack of professional recognition may raise the risk of frustration, as was described also by our participants, and potentially lead to burnout.^22^ Although the importance of interprofessional education and competencies related to working in healthcare teams has been emphasised for quite some time,^23^ it seems that the education system is still not equipping HCPs sufficiently with interprofessional competencies,^24^ as shown also in the Czech context, where graduating medical students had limited awareness of dietitians’ role or scope of practice.^8^

The internal differences in the understanding of dietitians’ role were noticeable particularly in the areas of education and job description, especially regarding the relevance of food service employment. Some dietitians viewed the combination of clinical and foodservice responsibilities as beneficial, emphasising the importance of integrating both areas rather than working in isolated silos. This view is similar to the opinion of dietitians in other studies.^25 26^ Others, however, perceived involvement in foodservice operations as less prestigious or unrelated to their professional role and preferred to focus solely on clinical care. This was documented by other researchers, especially in dietetic students, who felt they would not be able to demonstrate their competence in full.^27 28^ In the Czech context, this divide may partly reflect the legacy of the former system of dietetic nurses, whose competencies were strictly tied to foodservice operations. The shift towards a modern concept of AHPs may thus include a desire to distance the profession from this area, similar to what happened, for example, in the UK, during the 1960s, where clinical dietetics overshadowed food service role of dietitians.^29^ Educational institutions appear to reinforce this trend by emphasising clinical placements and competencies.^25 27^ This divergence reflects the need to redefine the role of dietitians in modern Czech healthcare for both education and practice and the need for further investigation of the professionalisation of dietitians in the Czech historical context and attitudes and practices of Czech education institutions regarding food service area.

### Missing culture of nutrition care

Despite the inclusion of medical nutrition therapy in the clinical guidelines of numerous health conditions^30 31^ and evidence supporting the benefits of nutrition care and dietitian involvement for optimal patient outcomes,^32^,^35^ many participants reported a lack of other staff interest in nutrition care. This resulted in insufficient resources, improper nutrition practice and poor recognition of dietitians. Our participants often considered these to be key reasons for suboptimal nutrition care, as did the participants of several other studies, who highlighted nutrition culture of the whole organisation as one of the essential conditions for functioning nutrition care.^13 36^

Current nutrition education for HCPs is not optimal. Students’ demand for nutrition knowledge is often confronted with a lack of academics educated in this area and rather isolated lectures outside the curriculum, leaving them dependent only on their interest in the topic and self-study of the literature.^37 38^ A recent assessment in Czechia^39^ found that nutrition education hours in medical school were below the recommended minimum by ESPEN,^40^ medical students felt unprepared to offer nutrition care, and their awareness of the role of nutrition in clinical practice was limited.^8^ This aligns also with findings of a systematic review on medical nutrition education, which identified that nutrition is not emphasised in medical curriculum, medical students feel insufficiently prepared, and they rarely interact with dietitians.^7^

Participants described the deprioritisation of nutrition care by nurses, reporting perceptions that they did not consider nutrition interventions important, lacked time for their administration or intentionally skipped nutrition screening or fabricated its results. Available research shows that nurses in general do not consider nutrition unimportant, but cope with other barriers, such as high workload, working in silos, unclear roles and low staffing, leading them to prioritise other activities.^3641^,^43^ With the nutrition screening specifically, lack of guidelines and proper equipment, and the complexity of some screening tools can further decrease the motivation.^44^ However, nurses themselves call for more education in this area, attaching greater importance to nutrition care with deeper knowledge.^4244^,^46^

Regarding hospital management, the last group mentioned in our interviews, participants described the reluctance to open new positions for dietitians, restrictions of their professional responsibilities and a lack of access to necessary equipment or medical records, all of which represent organisational barriers according to Wei *et al*, with the ability to impede interprofessional collaboration and quality nutrition care.^12 13^ Hospital management plays a key role in establishing the nutrition culture by providing sufficient resources, communicating the values and providing a sense of importance to the team members.^47^ However, their improper decisions can hamper the provision of nutrition care,^48^ as interprofessional collaboration can flourish only in an environment of institutional support, positive work culture and a suitable physical environment.^23^ Implementation-oriented activities focused on the support of both undergraduate education and continuous professional development are needed together with the evaluation of their impact. Further investigation of the management decision-making process could also help tailor other interventions to support the development of nutrition culture in the whole organisation.

### When the system holds the profession back

Participants expressed aspirations which currently do not fit into the Czech legal framework of the profession. While many participants advocated for greater autonomy in terms of ONS prescription or diet order adjustment, current legislation restricts these activities to physicians only, despite the evidence of effectiveness of the dietitians’ professional scope expansion.^49 50^ This lack of autonomy, which might reflect the underdevelopment of organisational professionalism,^20^ where lack of autonomy can hinder the consolidation of a strong professional identity, was described to lead not only to limited effectiveness of nutrition care, but also to feelings of professional inferiority. However, it remains debatable whether granting full autonomy in selected clinical tasks alone would resolve the issue. Since clinical decision-making suffers from the lack of effective interprofessional collaboration and communication,^51^ granting higher autonomy would have to go hand in hand with the support of teamwork in the hospitals. A broader discussion about legislative reform and clearer professional standards to align the system with the evolving role of dietitians in clinical care is needed to address these issues appropriately.

Together with other systemic issues such as low salaries, insufficient staffing and the absence of national guidelines for minimum dietetic coverage, all these issues might collectively hinder the development of organisational professionalism,^20^ preventing the profession from further growth. Furthermore, low staffing can negatively impact teamwork, productivity and staff turnover,^6 12^ which can in turn hinder the nutrition care offered and negatively impact patient outcomes. For example, according to Eglseer and Bauer,^52^ dietetic understaffing can be a major factor responsible for up to two-thirds of malnourished patients not receiving proper nutrition care. Setting a minimum level of dietetic staffing might benefit the entire nutrition care system in the Czech Republic.

## Limitations

This study provides valuable insight into the challenges faced by hospital dietitians in the Czech Republic; however, several limitations should be considered. The exclusive focus on dietitians’ perspectives may have resulted in a one-sided view of interprofessional collaboration, as the voices of other HCPs were not included. The recruitment strategy, relying on professional bodies and social media, may have favoured more engaged or dissatisfied individuals, introducing potential selection bias. The principal investigator’s professional role and visibility within the field could have influenced participants’ responses due to perceived authority. Additionally, while efforts were made to include diverse hospital types, some settings may have been underrepresented. Finally, the findings are context-specific and may not be generalisable to countries with different healthcare systems or professional structures.

## Conclusions

According to hospital dietitians participating in this study, there is limited awareness among other HCPs about dietitians’ role, leading to insufficient utilisation of nutrition to support clinical care. Moreover, dietitians themselves struggle to find a shared professional identity, which might hinder the further evolution of the profession. Lack of support from hospital management, limited resources, restricted autonomy and understaffing were perceived as contributing to frustration among dietitians and may complicate efforts to provide optimal nutrition care to patients. However, there are hospitals where dietitians reported successful integration into interdisciplinary teams. The findings indicate that improvements in legislation, education and hospital governance could support the evolving role of dietitians in Czech healthcare. Clarification of the professional role of dietitians, both in clinical and foodservice contexts, may help its further development together with reflecting this in undergraduate training and continuous professional development. Future research could explore the impact of these interventions and decision-making processes within hospital management to foster a stronger culture of nutrition care. This research should include longitudinal studies to capture the evolving role of dietitians and the long-term effects of systemic interventions.

## Supplementary material

10.1136/bmjopen-2025-101787 online supplemental file 1

## Data Availability

Data are available on reasonable request.
