# Supplementary material for: Challenges in delivering nutrition care perceived by hospital dietitians in the Czech Republic: a qualitative study
Source: BMJ Open. 2025 Nov 4;15(11):e101787. doi: 10.1136/bmjopen-2025-101787 (PMC12588016; doi:10.1136/bmjopen-2025-101787)
Supplement: online supplemental file 1 [file bmjopen-15-11-s001.docx]

**Supplementary table 1** Interview guide

| **Domain** | **Questions** |
| --- | --- |
| Demographic data | Gender, age, level of education in dietetics (incl. year of graduation), specialized education (incl. year of graduation), hospital name, years of practice, length of current employment, workload level (hrs/week), professional role |
| Job satisfaction | Please describe your job to me. What kind of work do you do, what are your duties?  What activity do you spend most of your working hours doing?  Please rate your overall job satisfaction.  What do you enjoy, find fulfilling about your work?  What demotivates you, what bothers you?  Please describe your working environment - your office, its location, technology, etc. How satisfied are you with it?  How satisfied are you with your salary? Describe how you find the salary relevant to your job, its demands and its workload. |
| Perception by other health professionals | How do you think your work is valued and accepted by other health professionals in your workplace?  Do you think there is a difference in the experiences of you and your colleagues?  What healthcare professionals or other staff do you interact with while caring for a particular patient?  Please describe how the interdisciplinary team works in your practice. How do you feel involved in the interdisciplinary team at your workplace and what is your role in it?  Please name the barriers to the dietitian’s full involvement in the team and ways you think they could be removed. |
| Perception of job description | To what extent does your job description match your expectations? If it does not match, what should ideally be different?  To what extent does your job description match your position?  How does your job description match your training in dietetics?  Please think about your workload and how you do or do not manage your tasks within your work hours. How do you perceive the staffing situation in providing nutrition care at your workplace? |
| Perception of nutrition care | What are the barriers in your workplace that prevent you from providing care as you would like to? Please describe them. What would need to change to improve the situation?  How do you see nutrition care in the Czech Republic? Describe what are the weak or strong points in your opinion and experience. |
