# Supplementary material for: Challenges in delivering nutrition care perceived by hospital dietitians in the Czech Republic: a qualitative study
Source: BMJ Open. 2025 Nov 4;15(11):e101787. doi: 10.1136/bmjopen-2025-101787 (PMC12588016; doi:10.1136/bmjopen-2025-101787)
Supplement: Supplementary file 2 [file bmjopen-2025-101787.reviewer_comments.pdf]

PEER REVIEW HISTORY

BMJ Open publishes all reviews undertaken for accepted manuscripts. Reviewers are asked to complete a checklist review form and are provided with free text boxes to elaborate on their assessment. These free text comments are reproduced below.

ARTICLE DETAILS

Title (Provisional)

Challenges in delivering nutrition care perceived by hospital dietitians in the Czech Republic: a qualitative study

Authors

Krobot, Martin; Jančeková, Kamila; Hawk, Victoria H; Zelenková, Veronika; Kapounová, Zlata

---

VERSION 1 - REVIEW

**Reviewer**	**1**
**Name**	**Evelina, Liljeberg,**
**Affiliation**	**Uppsala University, Food Studies, Nutrition and Dietetics**
**Date**	**08-May-2025**
**COI**	**None**

General comments

This manuscript explores an important topic—dietitians' experiences of the dietitians role and perceived barriers to effective nutrition care in acute care settings, with a focus on the Czech Republic. The manuscript could better address the significance of this setting in relation to international differences in the profession.

Key areas for improvement include:

- Introduction: Currently too brief and lacks a clear rationale for the study.
- Methods: The description of the research process is limited and needs more detail.
- Analysis: The thematic analysis appears underdeveloped and may reflect an unclear or inconsistent aim.

Despite these issues, the study has strong potential to contribute to the field of dietetics and the nutrition care process within healthcare.

Keywords

1. I'm unclear on the inclusion of "Decision Making" as a keyword. It doesn't appear to be a prominent theme in the manuscript—could the authors clarify its relevance?

Title

2. I like the title. However, I'm curious about the emphasis on "Interdisciplinary" - was this a

central focus of the study, or a more secondary theme?

Abstract

3. The abstract would benefit from including the number of participants and a brief mention of the analysis method (i.e., thematic analysis) to give readers a clearer overview of the study design.
4. The conclusion feels somewhat strong given the qualitative nature of the study and the relatively small sample size (15 dietitians). A more cautious interpretation would be more appropriate.

Introduction

5. The brevity of the introduction limits the strength of the argumentation regarding the existing knowledge gap and the rationale for conducting the study.
6. The manuscript would benefit from a stronger theoretical foundation. Key concepts—such as interdisciplinary care, nutrition care, and the dietetic profession in a global context—should be more clearly defined and situated within existing literature. Introducing a theoretical framework, such as professionalism or the development of professions (e.g., Evetts, J. Professions: Changes and continuities, International Review of Sociology, 1999;9:75–86), could enhance the conceptual clarity of the study. Alternatively, the authors might consider applying a framework that categorizes the barriers at different levels (e.g. individual, team, system), as mentioned in the discussion. If integrated into the analysis, such a framework could also deepen the interpretation of findings and strengthen the overall analytical depth.
7. The aim of the study is described inconsistently across the manuscript. In the introduction, it differs from the version presented in the first paragraph of the methods section. Was the aim to explore dietitians' experiences of "barriers to effective nutrition care," "barriers in multidisciplinary nutrition care," or something else? The former appears broader than the latter. Additionally, the opening of the discussion introduces yet another angle, stating that this is the first qualitative study exploring Czech dietitians' job satisfaction and perceptions of their role in providing nutrition care. This lack of clarity around the study's aim makes it difficult to interpret the findings with confidence.

Methods

8. Overall, the methodological section lacks grounding in established qualitative research literature. Strengthening this part with references to relevant methodological sources would enhance the credibility and transparency of the research process.
9. The authors use varying terms—such as convenience, purposive, and snowball sampling—to describe the sampling strategy in the abstract and methods section. This inconsistency makes it unclear which approach was actually employed. Please clarify the primary sampling method and support the description with appropriate references to methodological literature.
10. It is intriguing that the authors included a notetaker on Zoom—off camera—to take field notes during the interviews. Is this approach supported by existing methodological literature? It would be helpful to reflect on how the presence of an unseen observer might have influenced participants' comfort or responses. Additionally, in the ethics section, the authors state that only the principal investigator knew the participants' identities. However, it seems likely that the notetaker also had access to this information—could the authors clarify this point?
11. The description of the data analysis process is currently too brief. A more detailed account of how the analysis was conducted—particularly how themes were identified and refined—would strengthen the transparency and trustworthiness of the findings.

12. Under the “Patient and Public Involvement” section, it would be helpful to explicitly state that only dietetic professionals were involved in this study, and that there was no patient or public involvement.

Results

13. Do the three themes clearly address the stated aim of the study? As noted in my earlier comment (see point 7), the aim is somewhat inconsistently presented, which makes it difficult to assess alignment. I am particularly concerned about the third theme, “Self-perception of dietitians.” The findings in this theme appear to overlap with content from the other two themes and may not stand as a distinct category. Further clarification or reorganization might strengthen the thematic structure.
14. The titles of the themes and sub-themes could be refined to better reflect the content they represent. For instance, the theme “Nutrition knowledge” may not fully capture the range of issues discussed in that section. Is the focus solely on knowledge, or does it also involve a broader limited understanding of nutrition care or even a deprioritization of nutrition care?
15. The current theme names are closely tied to the data and remain at a largely semantic level. To enhance the analytical depth of the results section, consider clarifying the study’s aim, providing a more detailed description of the analytical process, and applying a theoretical framework. These steps could support a more interpretive approach to theme development and strengthen the overall contribution of the findings.
16. In Table 1, I recommend merging the columns for number and percentage, and presenting the percentage values in parentheses.

Discussion

17. The discussion section introduces several concepts and references that would have been more appropriately placed in the introduction. Including this background earlier would help establish a stronger context for the study and better support the rationale for the research.
18. I recommend using subheadings in the discussion that go beyond simply repeating the theme names. More interpretive or explanatory subheadings could help guide the reader and clarify the broader meaning and implications of the findings.
19. The tension between clinical dietetics and food service dietetics is a well-known issue. If you choose to retain this subtheme, I would suggest clarifying its relevance to your current aim. Additionally, it would strengthen the discussion to engage more directly with existing literature on this topic to contextualize your findings.

Conclusion

20. I really like the first sentence of your conclusion—it’s clear and well put. You might consider using it to help sharpen your aim and guide your analysis.

**Reviewer**	**2**
**Name**	**Iatridi, Vasiliki**
**Affiliation**	**Oxford Brookes University**
**Date**	**16-Jul-2025**
**COI**	**None**

General Comments

This is an interesting and valuable study with strong potential to contribute useful insights to the subject area and with findings that are relevant to a broad range of healthcare professionals.

That said, the current focus appears to lean more towards dietitians' perspectives on their professional identity and their position within MDTs, rather than directly addressing barriers to nutrition care. Barriers in nutrition care seem to emerge as a consequence of the concerns and challenges raised by the interviewed dietitians, rather than being the primary focus of the research as suggested by the manuscript title.

In addition, the research rationale and study aims, as presented in both the Abstract and Introduction, could benefit from clearer alignment. It would be helpful for readers to understand whether the interviews primarily explored barriers to nutrition care itself, or challenges with the dietitics profession that may in turn affect nutrition care provision. If the former is intended, a stronger justification for the research questions would be useful. If the latter is the case, then revisiting the manuscript title and study aims to reflect this more accurately would strengthen the overall clarity and coherence.

As illustrated in Table 1 (interview guide), only a small proportion of the questions directly explored barriers to nutrition care, with most focusing on dietitians' position within healthcare teams and related topics. While the themes and subthemes are framed under the 'barriers to nutrition care' narrative, much of the supporting argumentation and quotations provided appear to align with a different research emphasis. Similarly, the opening sentence of the Discussion section ("*To our knowledge, this is the first qualitative study of Czech dietitians in acute care settings exploring their job satisfaction and perceptions of their role in providing nutrition care*") reinforces this impression and may be worth reconsidering.

Finally, for improved readability, it would be beneficial to ensure consistent use of tense (either present or past) throughout the manuscript.

Abstract

- Line 30: It may be clearer to mention the analysis approach once, at the end of the Methods section.
- Line 31: For consistency and clarity among reading audiences working at different heathcare systems, please consider using the same terminology for the study participants (e.g. "acute care dietitians" or "hospital dietitians") and providing a brief definition in the manuscript, as different healthcare systems may classify acute dietitians differently. The same applies to what a chief or other named dietitian roles represent. In fact, in the Results section, it appears that not all dietitians who participated in this research held positions relevant to acute care. Likewise, in Lines 50-51, it is stated that dietitians from different healthcare settings were recruited. Later in the manuscript, participants are also referred to as clinical dietitians. Given the nature

of the research question, it is important to ensure clarity and avoid any potentially misleading information regarding the participants' area of work or specialty and where/to whom these findings can be relevant to.

- Kindly ensure the sample size is reported in either the Methods or the Results section.
- Line 34: [...] *other* healthcare professionals [...]
- It would be helpful if the themes were stated clearly in the Results section.
- It would strengthen the abstract to better align the suggested future research with the study findings.

- Line 57: The relevance of including limitations related to self-reported data and potential recall bias for this type of qualitative research methodology could be reconsidered.

Introduction

- Lines 68-69: The authors should expand their argument to highlight the gap in the literature: what is the difference between the barriers already identified and the aim of this research?

Methods

- Lines 72-78: The authors may wish to reconsider the placement of arguments related to topics covered in different subsections, i.e., recruitment, interview methods, qualitative analysis, and the presentation of results.
- Line 80 / Line 83 / Line 107 / Line 116: There are several references to purposeful or non-purposive recruitment that appear to include contradictory information. The authors are encouraged to review and clarify their description of the recruitment approaches.
- Lines 81-82 vs. Lines 114-116: Repetitive information – revise accordingly.
- Lines 93-95 vs. Lines 109-110: While it is stated that the note-takers were only present during the recorded part of the interviews, with recordings starting after demographic information was collected, it is not uncommon for participants to share identifiable information during qualitative interviews. Therefore, the statement that only the main interviewer was aware of the participants' identities may need to be reconsidered or clarified. In addition, it would be helpful to explain the purpose of the confidentiality agreements.
- Line 85: The characterisation of the interviews as "in-depth," given that the median duration was 36 minutes, may need to be reconsidered.

- Lines 88-90: While I appreciate the thorough preparation and pre-testing of the interview questions, peer-reviewed journals cannot publish data collected without adherence to the Declaration of Helsinki. The authors should either rerun their analysis excluding the pilot data or clarify and revise if these individuals were not “potential” participants but were in fact recruited and had data collected following standard informed consent procedures.
- It is not usual practice to include the full interview schedule in the main Methods section. Authors are advised to summarise the key topics or domains covered and/or provide a few example questions, with the full schedule placed in an appendix or supplementary material if needed.
- I would typically expect the statement on ethics to be discussed alongside the recruitment strategies and before data collection.

Results

- Lines 81-82 vs.115-116: Inocnsident reporting/explanation of the snowballing recruitement approach.
- Table 2
    - The female-male responses refer to sex assigned at birth rather than gender. The authors are encouraged to add a relevant footnote explaining the term used in the original language and any conventions applied during translation.
    - Any categories that include “</>” signs should be revised, as minimum and/or maximum values were also relevant in cases.
    - It is unclear how a high school of college degree are categories of “Level of dietetics education”. This classification also refers to highest level of the relevant education.
    - The percentages of the different professional roles needs to be presented or/and calculated differently.
- The subthemes under Theme 2 would benefit from revision, both for clarity (e.g., “ineffective system”) and for the use of more neutral and considerate language (e.g., “nonfunctional leadership”).
- It is difficult to understand the link between Theme 3 and the barries to nutrition care, while the names of its subthemes are also quite vague (“food service”) or need rephrasing for clarity and/or clearer link to the suthemes content (e.g. “education in dietetics”, “Professional respect and compensation”)
- Line 136: It is unclear what does nutrition refers to? Is it nutrition knowledge, nutritoon care?

- Lines 137, 145, 219 (and more examples throughout): Unless the authors are presenting word-for-word participant quotes, the language used throughout the remainder of the manuscript should be more considerate and respectful towards healthcare systems and the workforce. In addition, the authors are strongly encouraged to reflect on whether certain parts of the subthemes and the associated quotes represent isolated individual cases or unique instances of serious malpractice, rather than common themes or subthemes across participants. They are therefore advised to consider what value the scientific and clinical community would gain from including these in the research output.
- Lines 144-150: Example not relevant to lack of nutrition knowledge.
- Lines 152-154: Revision is required to provide a clearer link between the consequences described and the related subtheme. Overall, both quotes presented focus on limited budgets for dietetics departments and do not indicate that the participants associated these issues with a lack of nutrition knowledge.
- While subtheme 1.2 could potentially fit under Theme 1, much of the argumentation does not establish a clear link between the recognition of dietitians and the importance of nutrition. A similar concern applies to subtheme 1.3, where the link to the importance of nutrition seems to be a conclusion or assumption made by the researchers rather than a point explicitly argued by participants. The authors are advised to either select different quotes that demonstrate this link more clearly or reconsider the choice of themes and subthemes.
- Lines 185 & 197: Revision is required for clarity including the use of word 'facilities'.
- Lines 192-195: Revision is required for clarity and relevance to the linked subtheme and theme.
- Lines 217-218: [...] including the chief dietitian // [...] including chief dietitians
- Lines 228 vs. Line 232: It is unclear if the head of food service is a dietitian or not.
- Lines 234-236: The quote provided is not relevant to the stated subtheme and, in addition, focuses on sex and age/experience discrimination rather than dismissive behaviour related to professional identity.
- The 'scope of practice' is typically defined by the government and/or relevant regulatory bodies and practicing beyond one's scope of practice xconstutues breach of professional requirements and result in reporting. The autors are advised to reconsider whether participants expressed frustaryon with what their regulatory body permits tem to do or rephrase to ensure that the barrier it is hiw the scope of dietetic practice is ntereptres by other HCPs. If the profession is not regulated, a relevant backgroud information is required in the Intriduction where the context diettians work in hospitals is presented, what education is required for one to be empyed as dietians, etc. Some

paralles t the UK NHS system may help with furhe rclarity particularly cosndiering the target journal.

- Line 268: It is unclear whether this is a finding from the interviews.
- Selected quotes were relevant to Subtheme 2.3, but the argumentation about the challenges in accessing patient records was not linked to these quotes.
- Line 288: Revision is required for clarity.
- The argument in Lines 333–334 about an internal sense of lacking value, together with the quotes in Lines 335–338, does not align with the later shift toward external recognition instead.

Discussion/Conclusions

- Line 367: Dietitians are also considered staff.
- Line 370: Findings from one systematic review are discussed but there are two distinct in-text citations. Similar concern in Line 374 with two in-text citations for a single ESPEN position paper.
- Lines 378, 388-389, 391-392, 449: Revision is required for clarity.
- Line 384: Why is there an in-text citation when own research findings are reported?
- Line 385: Replace “on” with “in”
- Subtheme 1.3 is missing from the discussion of Theme 1 and appear in the discussion of Theme 2.
- Line 403: The argument regarding the benefits of a “clear job description” in relaton to improved nutrition care is incomplete/missing.
- Lines 404-405: The authors are advised to search the literature (e.g., https://dcjournal.ca/doi/10.3148/cjdpr-2018-018) and relevant regulatory or professional bodies resources as there is some evidence linking RD staffing with patient outcomes and staffing wellbeing.
- Line 443: Statement poorly supported by study findings.
- Line 444: The authors may wish to reconsider the statement suggesting that the limitations are inherent to the chosen qualitative research methodology, as this could risk undermining its broader value. Overall, this section would benefit from revision by providing stronger argumentation and ensuring that all concerns raised in previous sections are clearly and coherently outlined.
- Line 446: It is unclear where ‘sound methodology’ refer to.

- Lines 452-453: The direct link between the challenges and concerns raised by dietitians regarding their professional role and a potential threat to patients at risk of malnutrition is currently weak and should be toned down, as it is not an overarching finding across all themes in this research.
- The suggestions for future research are interesting; however, a study proposal or future directions more clearly linked to the study's findings could be added.

VERSION 1 - AUTHOR RESPONSE

Reviewer 1

Comment: *I'm unclear on the inclusion of "Decision Making" as a keyword. It doesn't appear to be a prominent theme in the manuscript—could the authors clarify its relevance?*

Reply: We agree, and we are aware of this issue. When submitting the original manuscript, we were asked to choose MeSH terms from a given list, which did not reflect the breadth of MeSH terms. It was stated by the publisher that if some terms were missing, they could be added throughout the review process. However, we were forced to choose a minimum number of MeSH terms among the offered, so we tried to choose at least the closest ones. We have changed the keywords using the full list of MeSH terms to reflect the actual content of the article and the refined analysis.

Comment: *I like the title. However, I'm curious about the emphasis on "Interdisciplinary" - was this a central focus of the study, or a more secondary theme?*

Reply: Thank you for pointing this out. You were right – the title was not reflecting the contents of the study accurately. After revising the aim and rerunning the analysis, we have adjusted the title to be more descriptive of the overarching aim of the study.

Comment: *The abstract would benefit from including the number of participants and a brief mention of the analysis method (i.e., thematic analysis) to give readers a clearer overview of the study design.*

Reply: We agree and we have added the number of participants. However, we are not sure about the recommendation on the analysis method, since applied thematic analysis has been already mentioned in the section. We have moved it to the end based on other comments; we hope this will make it more apparent as a standard position for the employed analysis method.

Comment: *The conclusion feels somewhat strong given the qualitative nature of the study and the relatively small sample size (15 dietitians). A more cautious interpretation would be more appropriate.*

Reply: Thank you for pointing this out. We have rewritten the conclusion according to the revised analysis and discussion and we believe we were more considerate about the language used.

Comment: *The brevity of the introduction limits the strength of the argumentation regarding the existing knowledge gap and the rationale for conducting the study.*

Reply: We agree and this is related to the original word limit. We have reworked the introduction to provide more context on the contents and rationale.

Comment: *The manuscript would benefit from a stronger theoretical foundation. Key concepts—such as interdisciplinary care, nutrition care, and the dietetic profession in a global context—should be more clearly defined and situated within existing literature. Introducing a theoretical framework, such as professionalism or the development of professions (e.g., Evetts, J. Professions: Changes and continuities, International Review of Sociology, 1999;9:75–86), could enhance the conceptual clarity of the study. Alternatively, the authors might consider applying a framework that categorizes the barriers at different levels (e.g. individual, team, system), as mentioned in the discussion. If integrated into the analysis, such a framework could also deepen the interpretation of findings and strengthen the overall analytical depth.*

Reply: Thank you for these recommendations. We have reworked the introduction to provide more context on the issue and we have employed the frameworks by Evetts and Wei, which you can find throughout the Introduction, Results and Discussion parts.

Comment: *The aim of the study is described inconsistently across the manuscript. In the introduction, it differs from the version presented in the first paragraph of the methods section. Was the aim to explore dietitians' experiences of "barriers to effective nutrition care," "barriers in multidisciplinary nutrition care," or something else? The former appears broader than the latter. Additionally, the opening of the discussion introduces yet another angle, stating that this is the first qualitative study exploring Czech dietitians' job satisfaction and perceptions of their role in providing nutrition care. This lack of clarity around the study's aim makes it difficult to interpret the findings with confidence.*

Reply: Thank you for bringing attention to this. We have revised and unified the declared aim of the study throughout the manuscript to challenges and experiences, which was the original aim of the study, since the study aimed to capture the general conditions of dietitians' day-to-day work. We believe this is now consistent with the results of the revised analysis.

Comment: *Overall, the methodological section lacks grounding in established qualitative research literature. Strengthening this part with references to relevant methodological sources would enhance the credibility and transparency of the research process.*

Reply: Yes, we have originally stated only the handbook of applied thematic analysis. After elaborating the methodological section, we have added sources supporting the sampling strategies and notetaker presence. At the same time, we did not want to add sources only to raise their number; therefore, we tried to distinguish between information coming from Guest's ATA handbook and information coming from other sources.

Comment: *The authors use varying terms—such as convenience, purposive, and snowball sampling—to describe the sampling strategy in the abstract and methods section. This inconsistency makes it unclear which approach was actually employed. Please clarify the primary sampling method and support the description with appropriate references to methodological literature.*

Reply: Thank you for pointing this out. We admit that we have misinterpreted the term "convenience sampling". We revised and elaborated the part about sampling, removing convenience sampling and replacing snowball sampling with chain referrals to better reflect the purpose of acquiring contacts for dietitians, who were meeting our predefined criteria. We have also added the literature clarifying the sampling methods.

Comment: *It is intriguing that the authors included a notetaker on Zoom—off camera—to take field notes during the interviews. Is this approach supported by existing methodological literature? It would be helpful to reflect on how the presence of an unseen observer might have influenced participants' comfort or responses. Additionally, in the ethics section, the authors state that only the principal investigator knew the participants' identities. However, it seems likely that the notetaker also had access to this information—could the authors clarify this point?*

Reply: As far as we know, there is no literature directly focusing on a notetaker as a third person during an online interview. However, we employed a third person as a form of researcher triangulation and at the same time to make the interviewer able to focus fully on the interview. We have elaborated on this in the revised methodology together with related literature. Regarding the notetaker's knowledge of participants' identities, we have added a comment on this in the data collection subsection, where we clarified that the notetaker did not have access to participants' personal data.

Comment: *The description of the data analysis process is currently too brief. A more detailed account of how the analysis was conducted—particularly how themes were identified and refined—would strengthen the transparency and trustworthiness of the findings.*

Reply: We agree; this was caused by the word limit of previous BMJ submission. We have elaborated the analysis part in the revised Methods section.

Comment: *Under the "Patient and Public Involvement" section, it would be helpful to explicitly state that only dietetic professionals were involved in this study, and that there was no patient or public involvement.*

Reply: Thank you for this recommendation. We have revised the PPI statement completely due to previous misunderstanding.

Comment: *Do the three themes clearly address the stated aim of the study? As noted in my earlier comment (see point 7), the aim is somewhat inconsistently presented, which makes it difficult to assess alignment. I am particularly concerned about the third theme, "Self-perception of dietitians." The findings in this theme appear to overlap with content from the other two themes and may not stand as a distinct category. Further clarification or reorganization might strengthen the thematic structure.*

Reply: Thank you for this comment. Based on both the reviews, we have rerun the analysis in the context of stated frameworks and we have introduced new themes and subthemes, which we believe explain much better what the participants were describing and are also better in alignment with the refined aim of the study.

Comment: *The titles of the themes and sub-themes could be refined to better reflect the content they represent. For instance, the theme "Nutrition knowledge" may not fully capture the range of issues discussed in that section. Is the focus solely on knowledge, or does it also involve a broader limited understanding of nutrition care or even a deprioritization of nutrition care?*

Reply: Yes, we agree. We changed the theme structure completely, but we tried to keep this in mind and to choose the names appropriately to encompass the issues covered by the theme.

Comment: *The current theme names are closely tied to the data and remain at a largely semantic level. To enhance the analytical depth of the results section, consider clarifying the study's aim, providing a more detailed description of the analytical process, and applying a theoretical framework. These steps could support a more interpretive approach to theme development and strengthen the overall contribution of the findings.*

Reply: Thank you for these recommendations. We believe that we have implemented all of them and that, as stated above, we are now presenting a more analytical version of the results better reflecting on the content of the interviews.

Comment: *In Table 1, I recommend merging the columns for number and percentage, and presenting the percentage values in parentheses.*

Reply: Thank you for this recommendation. We tried merging the columns as suggested, but after team discussion, we felt the original format was clearer for readers. We hope this decision is acceptable.

Comment: *The discussion section introduces several concepts and references that would have been more appropriately placed in the introduction. Including this background earlier would help establish a stronger context for the study and better support the rationale for the research.*

Reply: We agree. We have moved all the contextual information to the elaborated Introduction section.

Comment: *I recommend using subheadings in the discussion that go beyond simply repeating the theme names. More interpretive or explanatory subheadings could help guide the reader and clarify the broader meaning and implications of the findings.*

Reply: Thank you for this recommendation. We are now presenting subheadings, which we believe are interpretive, but still grounded in the data.

Comment: *The tension between clinical dietetics and food service dietetics is a well-known issue. If you choose to retain this subtheme, I would suggest clarifying its relevance to your current aim. Additionally, it would strengthen the discussion to engage more directly with existing literature on this topic to contextualize your findings.*

Reply: Thank you for pointing this out. The tension between clinical and food service dietetics is a part of a much broader issue with unclear professional identity of Czech dietitians. As such, we have refined the discussion on this topic and included relevant literature.

Comment: *I really like the first sentence of your conclusion—it's clear and well put. You might consider using it to help sharpen your aim and guide your analysis.*

Reply: We appreciate your feedback and included this sentence while refining the study's aim and guiding the final structure of the analysis. We believe that we have added more depth to the analysis and that we refined the aim to correspond with the research.

Reviewer 2

Comment: *Line 30: It may be clearer to mention the analysis approach once, at the end of the Methods section.*

Reply: We agree and we have moved the analysis approach to the end of the Methods section.

Comment: *Line 31: For consistency and clarity among reading audiences working at different heathcare systems, please consider using the same terminology for the study participants (e.g. "acute care dietitians" or "hospital dietitians") and providing a brief definition in the manuscript, as different healthcare systems may classify acute dietitians differently. The same applies to what a chief or other named dietitian roles represent. In fact, in the Results section, it appears that not all dietitians who participated in this research held positions relevant to acute care. Likewise, in Lines 50-51, it is stated that dietitians from different healthcare settings were recruited. Later in the manuscript, participants are also referred to as clinical dietitians. Given the nature of the research question, it is important to ensure clarity and avoid any potentially misleading information regarding the participants' area of work or specialty and where/to whom these findings can be relevant to.*

Reply: Thank you for this recommendation. To clarify the participants' background, we unified the terminology to "hospital dietitians" since this is the most suitable term regarding the sample. We have also clarified the recruitment section of Methods to; the original term "different healthcare settings" was meant as different types of hospitals and wards, which was not the most suitable term to use. We hope the revised version provides sufficient clarity on this point.

Comment: *Kindly ensure the sample size is reported in either the Methods or the Results section.*

Reply: Thank you for pointing this out. We have added the sample size to the Methods section.

Comment: *Line 34: [...] other healthcare professionals [...]*

Reply: Corrected.

Comment: *It would be helpful if the themes were stated clearly in the Results section.*

Reply: We have stated the themes in the Results section as suggested.

Comment: *It would strengthen the abstract to better align the suggested future research with the study findings.*

Reply: We have revised the future research directions and corrected them accordingly, together with aligning them better with the new version of Discussion.

Comment: *Line 57: The relevance of including limitations related to self-reported data and potential recall bias for this type of qualitative research methodology could be reconsidered.*

Reply: We agree and we have omitted this point from the section.

Comment: *Lines 68-69: The authors should expand their argument to highlight the gap in the literature: what is the difference between the barriers already identified and the aim of this research?*

Reply: Thank you for pointing this out. Together with the elaboration of the whole Introduction section, we have added more context on the main topics and also on the research gap.

Comment: *Lines 72-78: The authors may wish to reconsider the placement of arguments related to topics covered in different subsections, i.e., recruitment, interview methods, qualitative analysis, and the presentation of results.*

Reply: We have elaborated and restructured the Methods section based on your other comments and the comments of the second reviewer, including moving these to the suitable subsections.

Comment: *Line 80 / Line 83 / Line 107 / Line 116: There are several references to purposeful or non-purposive recruitment that appear to include contradictory information. The authors are encouraged to review and clarify their description of the recruitment approaches.*

Reply: Thank you for bringing attention to this. We admit that we have misinterpreted the term "convenience sampling". We revised and elaborated the part about sampling, removing convenience sampling and replacing snowball sampling with chain referrals to better reflect the purpose of acquiring contacts for dietitians, who were meeting our predefined criteria. We have also added the literature clarifying the sampling methods. We believe the sampling process is now clear.

Comment: *Lines 81-82 vs. Lines 114-116: Repetitive information – revise accordingly.*

Reply: Yes, we agree. The PPI statement was revised completely due to misunderstanding.

Comment: *Lines 93-95 vs. Lines 109-110: While it is stated that the note-takers were only present during the recorded part of the interviews, with recordings starting after demographic information was collected, it is not uncommon for participants to share identifiable information during qualitative interviews. Therefore, the statement that only the main interviewer was aware of the participants' identities may need to be reconsidered or clarified. In addition, it would be helpful to explain the purpose of the confidentiality agreements.*

Reply: Thank you for this comment. We have elaborated and reflected on the presence of notetaker and the potential knowledge of participants' identities. We have also added a note on the purpose of confidentiality agreements.

Comment: *Line 85: The characterisation of the interviews as "in-depth," given that the median duration was 36 minutes, may need to be reconsidered.*

Reply: As far as we understand this concern, we respectfully note that in most literature, in-depth interviews are characterized by their qualities, not the length as the only parametre (e.g. Rutledge, 10.1002/9781119011071.iemp0019). Jamshed (10.4103/0976-0105.141942) states that in-depth interviews take usually from 30 minutes to more than an hour. Although the median length of our interviews was 36 minutes, there were only 4 interviews shorter than 30 minutes. We have reviewed the interviews and these were interviews with participants who answered rather in short statements and did not respond well to any prompts seeking for deeper understanding of their perceptions. However, we still believe that the data from these interviews were a valuable asset to our analysis.

Comment: *Lines 88-90: While I appreciate the thorough preparation and pre-testing of the interview questions, peer-reviewed journals cannot publish data collected without adherence to the Declaration of Helsinki. The authors should either rerun their analysis excluding the pilot data or clarify and revise if these individuals were not "potential" participants but were in fact recruited and had data collected following standard informed consent procedures.*

Reply: We are sorry for the misunderstanding. Our pilot participants followed standard procedures including the informed consent. We have clarified this in the Methods section.

Comment: *It is not usual practice to include the full interview schedule in the main Methods section. Authors are advised to summarise the key topics or domains covered and/or provide a few example questions, with the full schedule placed in an appendix or supplementary material if needed.*

Reply: Thank you for this recommendation. We have provided example questions and moved the whole interview guide to a supplementary table.

Comment: *I would typically expect the statement on ethics to be discussed alongside the recruitment strategies and before data collection.*

Reply: Thank you for this recommendation. We have moved the ethics considerations accordingly.

Comment: *Lines 81-82 vs.115-116: Inocnsident reporting/explanation of the snowballing recruitement approach.*

Reply: Thank you for pointing this out. We have substantially reworked the Methods section, together with the sampling description. The PPI statement was changed completely and we have replaced snowball sampling with chain referrals to emphasize it was a means of getting new contacts based on predefined criteria rather than a sampling method. We believe that we have described this now clearly in the Methods section.

Comment: *The female-male responses refer to sex assigned at birth rather than gender. The authors are encouraged to add a relevant footnote explaining the term used in the original language and any conventions applied during translation.*

Reply: You are right. We have replaced the term Gender with Sex and added a footnote explaining the meaning and original word in Czech.

Comment: *Any categories that include “</>” signs should be revised, as minimum and/or maximum values were also relevant in cases.*

Reply: We have stated all the minimum and maximum values of the characteristics.

Comment: *It is unclear how a high school of college degree are categories of “Level of dietetics education”. This classification also refers to highest level of the relevant education.*

Reply: Thank you for this comment. We have addressed this in the Introduction section stating all the levels of education in dietetics which are relevant to Czech healthcare system. We have also specified the row title.

Comment: *The percentages of the different professional roles needs to be presented or/and calculated differently.*

Reply: Thank you for this recommendation. Since the percentages are meant to inform the reader about how prevalent was each possible role in the sample, we could not calculate them differently. We have added a footnote explaining what the percentages represent and that they do not add up to 100%.

Comment: *The subthemes under Theme 2 would benefit from revision, both for clarity (e.g., “ineffective system”) and for the use of more neutral and considerate language (e.g., “nonfunctional leadership”).*

Reply: Since we have revised the analysis completely, these subthemes are no longer present in the results. However, we have kept your comment in mind when choosing the names for new thematic structure.

Comment: *It is difficult to understand the link between Theme 3 and the barries to nutrition care, while the names of its subthemes are also quite vague (“food service”) or need rephrasing for clarity and/or clearer link to the suthemes content (e.g. “education in dietetics”, “Professional respect and compensation”)*

Reply: Since we have revised the analysis completely, this theme is no longer present in the results. However, we have implemented the contents into new themes and subthemes, since they were mostly linked to the professional identity or the legal boundaries.

Comment: *Line 136: It is unclear what does nutrition refers to? Is it nutrition knowledge, nutritoon care?*

Reply: This formulation was related to nutrition care. We have amended the formulation.

Comment: *Lines 137, 145, 219 (and more examples throughout): Unless the authors are presenting word-for-word participant quotes, the language used throughout the remainder of the manuscript should be more considerate and respectful towards healthcare systems and the workforce. In addition, the authors are strongly encouraged to reflect on whether certain parts of the subthemes and the associated quotes represent isolated individual cases or unique instances of serious malpractice, rather than common themes or subthemes across participants. They are therefore advised to consider what value the scientific and clinical community would gain from including these in the research output.*

Reply: Thank you for this recommendation. When reviewing the analysis, we tried to keep this in mind and distinguish more properly between the participants' perceptions and our interpretations.

Comment: *Lines 144-150: Example not relevant to lack of nutrition knowledge.*

Reply: Thank you for this comment. Although some participants attributed the suboptimal handling of nutrition screening to lack of knowledge about nutrition and consequences of malnutrition, it is true that this is not a directly linked phenomenon. We have newly made the issues of nutrition screening part of a broader theme of deprioritization of nutrition care, which we believe better captures the concept.

Comment: *Lines 152-154: Revision is required to provide a clearer link between the consequences described and the related subtheme. Overall, both quotes presented focus on limited budgets for dietetics departments and do not indicate that the participants associated these issues with a lack of nutrition knowledge.*

Reply: Similarly to previous comment, we have now moved the managerial issues under the deprioritization of nutrition care, which encompasses more than just nutrition knowledge and furthermore reflects the organizational barriers according to Wei.

Comment: *While subtheme 1.2 could potentially fit under Theme 1, much of the argumentation does not establish a clear link between the recognition of dietitians and the importance of nutrition. A similar concern applies to subtheme 1.3, where the link to the importance of nutrition seems to be a conclusion or assumption made by the researchers rather than a point explicitly argued by participants. The authors are advised to either select different quotes that demonstrate this link more clearly or reconsider the choice of themes and subthemes.*

Reply: We agree and we believe the new structure of themes and subthemes and the related argumentation have mitigated this issue.

Comment: *Lines 185 & 197: Revision is required for clarity including the use of word 'facilities'.*

Reply: The Results section has been rewritten. However, we have kept in mind to avoid unclear formulations connected with the word facility.

Comment: *Lines 192-195: Revision is required for clarity and relevance to the linked subtheme and theme.*

Reply: The Results section has been rewritten. This issue has been moved under the professional identity and role clarity theme and the formulation was revised.

Comment: *Lines 217-218: [...] including the chief dietitian // [...] including chief dietitians*

Reply: During the revision of the analysis, we have discussed this issue in the context of new themes and subthemes and considered this to be too anecdotal to include, in line with one of your previous comments. Thus, we have omitted the references to the chief dietitian.

Comment: *Lines 228 vs. Line 232: It is unclear if the head of food service is a dietitian or not.*

Reply: The Results section has been rewritten. This issue has been viewed in the context of newly identified themes and in line with the participants' statements considered to be part of the marginalization of nutrition care. The text has been adjusted and this formulation is missing.

Comment: *Lines 234-236: The quote provided is not relevant to the stated subtheme and, in addition, focuses on sex and age/experience discrimination rather than dismissive behaviour related to professional identity.*

Reply: We agree. The quote is not a part of the manuscript anymore and more proper quotes were selected instead.

Comment: *The 'scope of practice' is typically defined by the government and/or relevant regulatory bodies and practicing beyond one's scope of practice xconstutues breach of professional requirements and result in reporting. The autors are advised to reconsider whether participants expressed frustaryon with what their regulatory body permits tem to do or rephrase to ensure that the barrier it is hiw the scope of dietetic practice is ntereptres by other HCPs. If the profession is not regulated, a relevant backgroud information is required in the Intriduction where the context diettians work in hospitals is presented, what education is required for one to be empyed as dietians, etc. Some paralles t the UK NHS system may help with furhe rclarity particularly cosndiering the target journal.*

Reply: Thank you for this comment. The scope of practice in our article really means the one which is defined by legislation. After the revision of the analysis, this was identified as a separate theme describing the tension between the ambitions of dietitians and the boundaries which are set by the system. We believe that this clarifies this issue. Furthermore, we have

mentioned UK dietitians and their increasing autonomy in the Introduction section as a comparison to Czech dietitians.

Comment: *Line 268: It is unclear whether this is a finding from the interviews.*

Reply: The Results section was rewritten. Although this was a finding from several interviews, in the end, we treated this information as a detail of a more prominent issue of managerial deprioritization of dietitians' needs, since according to our respondents, these problems were usually unsuccessfully raised with the management. Thus, we have omitted this particular information from the Results.

Comment: *Selected quotes were relevant to Subtheme 2.3, but the argumentation about the challenges in accessing patient records was not linked to these quotes.*

Reply: Thank you for pointing this out. You are right, that the quotes were formely linked more to the resistance for change, which did not constitute the majority of the text. This part of the text is no longer present in the Results section and the content was merged with related issues under the new themes and subthemes.

Comment: *Line 288: Revision is required for clarity.*

Reply: The Results section was rewritten. Since this was a finding from a few interviews, in the end, we treated this information as a detail of a more prominent issue of dietetic education and professional identity. Thus, we have omitted this particular information from the Results.

Comment: *The argument in Lines 333–334 about an internal sense of lacking value, together with the quotes in Lines 335–338, does not align with the later shift toward external recognition instead.*

Reply: We are sorry for a misunderstanding, this was a language mistake stemming from Czech. We hope the part of new Results section on compensation provides sufficient clarity.

Comment: *Line 367: Dietitians are also considered staff.*

Reply: The Discussion section was rewritten and this particular formulation was not preserved. However, we checked also for all the other instances of referring to other HCPs and made amendments accordingly.

Comment: *Line 370: Findings from one systematic review are discussed but there are two distinct in-text citations. Similar concern in Line 374 with two in-text citations for a single ESPEN position paper.*

Reply: Thank you for pointing this out. On line 370, it was a remnant after sentence editing and was deleted during rewriting the Discussion section. On line 374, the citations belong to ESPEN

position paper and the Czech assessment of nutrition education at a medical school. The citations were separated and placed more clearly. We reviewed all citation placements to ensure clarity and consistency throughout the manuscript.

Comment: *Lines 378, 388-389, 391-392, 449: Revision is required for clarity.*

Reply: The Discussion part was rewritten and these formulations were not preserved.

Comment: *Line 384: Why is there an in-text citation when own research findings are reported?*

Reply: Thank you for this comment. Although originally the citation was meant to be a link to the dietetics history in the Czech Republic, you are right, that on second thought, it did not make sense. However, since the Discussion section has been rewritten, this formulation is no longer present in the manuscript.

Comment: *Line 385: Replace "on" with "in"*

Reply: Thank you, we have corrected all the instances of healthcare teams in the text.

Comment: *Subtheme 1.3 is missing from the discussion of Theme 1 and appear in the discussion of Theme 2.*

Reply: The Discussion section has been rewritten according to the new structure of Results. We kept your comment in mind and believe that the structure of Discussion is currently clear.

Comment: *Line 403: The argument regarding the benefits of a "clear job description" in relaton to improved nutrition care is incomplete/missing.*

Reply: We agree, that this issue could be argued more thoroughly. We believe this issue is currently encompassed by the notion of professional identity and organizational professionalism.

Comment: *Lines 404-405: The authors are advised to search the literature (e.g., https://dcjournal.ca/doi/10.3148/cjdpr-2018-018) and relevant regulatory or professional bodies resources as there is some evidence linking RD staffing with patient outcomes and staffing wellbeing.*

Reply: Thank you very much for the recommendation. After the revision of the analysis, we realized that discussing this issue is out of scope of the article. In the new Discussion section, we focused more on the profession itself and the perceptions of the participants without attempts to "objectivize the truth". Therefore, this part of the discussion has been omitted.

Comment: *Line 443: Statement poorly supported by study findings.*

Reply: Yes, we agree. We have aligned the statement with the refined study aim.

Comment: *Line 444: The authors may wish to reconsider the statement suggesting that the limitations are inherent to the chosen qualitative research methodology, as this could risk undermining its broader value. Overall, this section would benefit from revision by providing stronger argumentation and ensuring that all concerns raised in previous sections are clearly and coherently outlined.*

Reply: Thank you for this comment. After a team discussion, we have completely revised the Limitations subsection. We believe it is now more appropriate.

Comment: *Line 446: It is unclear where 'sound methodology' refer to.*

Reply: The Limitations have been rewritten and this formulation was not preserved.

Comment: *Lines 452-453: The direct link between the challenges and concerns raised by dietitians regarding their professional role and a potential threat to patients at risk of malnutrition is currently weak and should be toned down, as it is not an overarching finding across all themes in this research.*

Reply: Thank you for this suggestion. We have rewritten and reframed the Conclusions and chose the tone of the statements more considerately.

Comment: *The suggestions for future research are interesting; however, a study proposal or future directions more clearly linked to the study's findings could be added.*

Reply: Yes, we agree. We have aligned the future research directions with the new version of Results and Discussion to correspond better with the findings and added more specific suggestions.

VERSION 2 - REVIEW

**Reviewer**	**1**
**Name**	**Evelina, Liljeberg,**
**Affiliation**	**Uppsala University, Food Studies, Nutrition and Dietetics**
**Date**	**06-Sep-2025**
**COI**	

Congratulations on the revised version of your manuscript. You have provided satisfactory responses to the points raised in my previous review. The article has improved significantly. Given the extent of the revisions, I still have some additional feedback for your consideration.

Title

1. The revised title is much improved. However, it would benefit from greater specificity regarding the context of the challenges. Are the challenges related to working as a dietitian, delivering nutrition care, or another aspect of practice? I also believe the aim of the study could be clarified further in relation to this issue. Please see my detailed comments below.

Introduction

2. The introduction is well written. I have two minor comments. The stated aim of the study is “to explore the challenges faced by Czech dietitians working in a hospital setting.” It might be helpful to clarify what kind of challenges are being referred to. For example, you could specify whether these are e.g. “challenges working with nutrition care” or ”working challenges” or “challenges in delivering nutrition care”.
3. Line 114-123: I really appreciate that you chose to relate your findings to established frameworks—this significantly enhances both the quality and transferability of your results. However, when I read that the challenges were examined through the lens of these frameworks, I initially expected the analysis itself to be guided by them. In the current version, the frameworks are primarily used to discuss and interpret the findings (in the Discussion), while the analysis appears to have been conducted more inductively. It might be helpful to clarify this distinction in the text to better align the reader’s expectations with your methodological approach. For example, you could consider moving the text from lines 114– 123 to the Methods section—specifically, placing it at the end of the Data Analysis subsection. There, you could explain that while the analysis was conducted inductively, the findings will later be interpreted in light of the selected frameworks. Alternatively, you could simply revise the wording in the current section to make this clearer.

Result

4. The Results section is clear and engaging. I have one minor comment: the quote on lines 347–349 is hard to understand and doesn’t add much clarity. I suggest removing it or replacing it with a clearer example.

Discussion

5. Well done—the discussion is very well written. I suggest expanding the first sentence to clarify what the challenges specifically relate to.

VERSION 2 - AUTHOR RESPONSE

Comment: *The revised title is much improved. However, it would benefit from greater specificity regarding the context of the challenges. Are the challenges related to working as a dietitian, delivering nutrition care, or another aspect of practice?*

Reply: We specified the title according to the refined aim of study. Please, see below.

Comment: *I also believe the aim of the study could be clarified further in relation to this issue*.

Reply: Yes, we agree. We addressed this issue in the next comment, please, see below.

Comment: *The stated aim of the study is "to explore the challenges faced by Czech dietitians working in a hospital setting." It might be helpful to clarify what kind of challenges are being referred to. For example, you could specify whether these are e.g. "challenges working with nutrition care" or "working challenges" or "challenges in delivering nutrition care".*

Reply: Thank you for pointing this out. Since the article focuses on the challenges in terms of the conflict between wanting to provide the best care possible and barriers which prevent the dietitians from doing so, we agreed on using "challenges in delivering nutrition care" as it aligns the best with the core aim of the study. We also refined the abstract introduction accordingly.

Comment: *Line 114-123: I really appreciate that you chose to relate your findings to established frameworks—this significantly enhances both the quality and transferability of your results. However, when I read that the challenges were examined through the lens of these frameworks, I initially expected the analysis itself to be guided by them. In the current version, the frameworks are primarily used to discuss and interpret the findings (in the Discussion), while the analysis appears to have been conducted more inductively. It might be helpful to clarify this distinction in the text to better align the reader's expectations with your methodological approach. For example, you could consider moving the text from lines 114–123 to the Methods section—specifically, placing it at the end of the Data Analysis subsection. There, you could explain that while the analysis was conducted inductively, the findings will later be interpreted in light of the selected frameworks. Alternatively, you could simply revise the wording in the current section to make this clearer.*

Reply: Thank you very much for this recommendation! After a discussion, we chose to move this text to the end of data analysis part of Methods, where we understand that as a part of a comprehensive explanation of Methods given all in one place. We adjusted the text accordingly to follow the preceding text in the section.

Comment: *I have one minor comment: the quote on lines 347–349 is hard to understand and doesn't add much clarity. I suggest removing it or replacing it with a clearer example.*

Reply: We understand and after second reading, we agree that the quote might be unclear. We replaced the quote with another one, pointing out a particular example of deprioritization by management.

Comment: *The discussion is very well written. I suggest expanding the first sentence to clarify what the challenges specifically relate to.*

Reply: We adjusted the wording according to the refined aim stated in the Introduction.
